# The disease burden of respiratory syncytial virus in older adults

**DOI:** 10.1097/QCO.0000000000001000

**Published:** 2024-01-10

**Authors:** Sebastien Kenmoe, Harish Nair

**Affiliations:** a Centre for Global Health, Usher Institute, University of Edinburgh, Edinburgh, UK; b School of Public Health, Nanjing Medical University, Nanjing, Jiangsu, China; c School of Public Health, University of the Witwatersrand, South Africa

**Keywords:** disease burden, older adults, respiratory syncytial virus, vaccine

## Abstract

**Purpose of review:**

To highlight the respiratory syncytial virus (RSV) disease burden and the current developments and challenges in RSV prevention for older adults ≥60 years through analysis of RSV epidemiology and the effectiveness of emerging vaccines.

**Recent findings:**

In industrialized countries, RSV incidence rates and hospitalization rates among older adults are estimated to be 600.7 cases per 100 000 person-years and 157 hospitalizations per 100 000 person-years, respectively. Yet, accurately determining RSV morbidity and mortality in older adults is challenging, thus resulting in substantially under-estimating the disease burden. The in-hospital fatality rates vary substantially with age and geographies, and can be as high as 9.1% in developing countries. Two promising RSV vaccines for the elderly have been approved, demonstrating efficacies of up to 94.1%, signifying considerable advancement in RSV prevention. However, concerns over potential side effects remain.

**Summary:**

RSV is associated with a significant burden in older adults. While the landscape of RSV prevention in older adults is promising with the licensure of vaccines from two companies, current trial data underscore the need for additional studies. Addressing the real-world effectiveness of these vaccines, understanding potential rare side effects, and ensuring broad inclusivity in future trials are crucial steps to maximize their potential benefits.

## INTRODUCTION

Respiratory syncytial virus (RSV), first identified by Robert Chanock in 1956, is a single stranded RNA virus. RSV is a common respiratory pathogen and almost all children are infected by the age of 3 years [1]. RSV is a member of the Pneumoviridae family, characterized by enveloped viral particles. This envelope contains key surface glycoproteins such as the G protein, which allows the virus to attach to and enter host cells and stimulates neutralizing antibodies [2]. The F protein, another crucial component, facilitates attachment and fusion to the host cell membrane. The F protein also undergoes a shift from a prefusion to a postfusion form and remains a central target for vaccine and passive prophylactic measures development [3]. There are two subgroups – A and B. RSV is transmitted primarily through droplets released by coughing and sneezing and involves direct contact with infected persons and contact with contaminated surfaces. Some infants may remain contagious for longer and those with immunocompromised status may remain contagious up to 4 weeks [4]. RSV exhibits distinct seasonality in most parts of the world [5] and causes severe disease in the very young (infants <1 year) and the older adults ≥60 years [6,7^▪^,^8^^▪▪^]. 

**Box 1:**
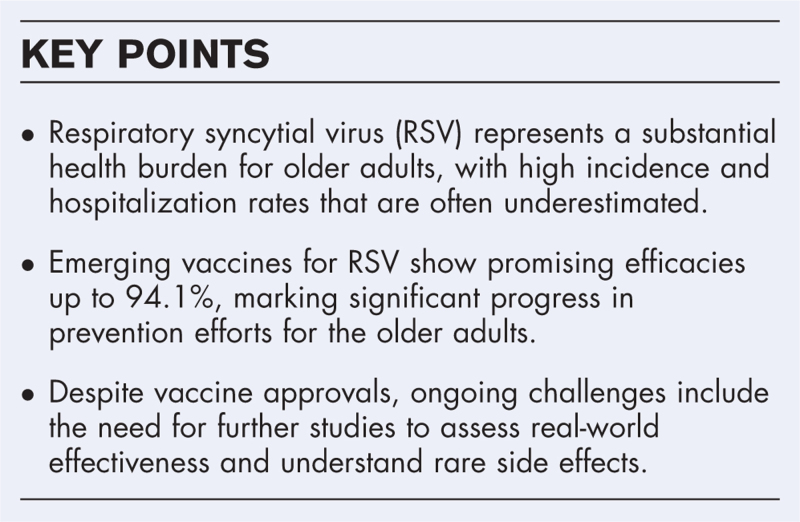
no caption available

RSV infection can result in upper respiratory and/or lower respiratory tract symptoms. The most prevalent upper respiratory tract infection (URTI) symptoms in older adults with RSV include sore throat, runny noses, and nasal congestion (Table 1). In those with lower respiratory tract infections (LRTI) symptoms, cough is predominant, along with shortness of breath and sputum. Among the gastrointestinal symptoms, nausea, vomiting, or diarrhea have been reported. Other common symptoms include fever, fatigue or weakness, disturbed sleep, and general malaise. The symptoms are mild and self- resolving in most cases with symptoms usually resolving in one to two weeks. However, a small proportion of cases may result in more severe acute LRTI leading to serious outcomes such as hospitalizations and death [7^▪^,^8^^▪▪^]. The incidence rates, however, show marked variability in any given year (across regions and countries) due to factors like inadequate testing and the potentially suboptimal sensitivity of conventional diagnostic specimens and tools in adults [9^▪▪^,^10^^▪▪^]. There is also marked variability in RSV activity year on year [5]. People with chronic conditions such as chronic obstructive pulmonary disease, congestive heart failure, diabetes mellitus, among others, are more likely to experience hospitalizations and death caused by RSV [11–13]. This vulnerability extends to those living in long-term care facilities, individuals displaying signs of frailty, those above 75 years of age, and immunocompromised patients, including organ transplant recipients [7^▪^]. The preventive and therapeutic options against RSV have remained limited until recently when effective immunization strategies have been developed [14^▪▪^,^15^^▪▪^]. The purpose of this article is to examine burden of disease caused by RSV in older adults (60 years and above).

**Table 1:** Respiratory syncytial virus signs and symptoms in older adults

	Mean proportion [range]
Upper respiratory tract infection symptoms	
Runny nose/rhinorhea [1–4]	58.5 [27.7; 81.8]
Nasal congestion [2,3]	56.4 [47.2; 72.7]
Sore throat/pharyngitis [1–5]	53.9 [23.7; 81.3]
Earache [2,4]	34.4 [32.3; 36.4]
Lower respiratory tract infection symptoms	
Cough [1–6]	78.9 [27.3; 97.2]
Sputum production [1–3,5,6]	72.8 [49.4; 94.4]
Shortness of breath/difficulty breathing/dyspnea [1,3–6]	66.9 [19.3; 89.1]
Hemoptysis [3,5]	1.8 [1.2; 2.9]
Chest pain [3,5]	21.1 [7.4; 34.9]
Wheezing [1–6]	55.8 [16.0; 55.8]
Tachypnea [3,5,6]	52.2 [6.6; 93.7]
Decreased breath sounds [3,5]	24.1 [7.8; 33.6]
Crackles/rales [3,5]	33.3 [9.5; 50.0]
Rhonchi [3,5]	33.2 [12.8; 44.4]
SpO2 <95% [6]	13.9
Pleuritic pain/pleuritic chest pain [3]	9.1 [6.8; 11.3]
Gastrointestinal symptoms	
Nausea/vomiting/ diarrhea [3,4]	28.4 [15.5; 38.7]
Anorexia/lack of appetite [3,4]	37.5 [22.7; 61.3]
Abdominal pain [3]	12.7 [12.4; 13.0]
Other symptoms	
Fever [1,3–6]	35.8 [5.6; 71.0]
Feverishness [1–3,6]	44.1 [27.3; 59.7]
Chills [3]	38.7 [35.7; 41.6]
Myalgia/muscle ache [1–6]	35.3 [16.0; 64.1]
Arthralgia/joint pains [3]	13.6 [13.0; 14.1]
Headache [1–4,6]	52.5 [16.7; 82.1]
Fatigue/weakness [1,3,4]	68.1 [47.9; 90.3]
Lethargy [3]	56.1 [54.5; 57.6]
Altered mental status [3,4]	32.2 [19.3; 40.0]
Disturbed sleep [6]	72.2
Feeling unwell [6]	91.7
Disturbance in daily activity [6]	75.0
Seizures [3]	1.2 [0.2; 2.1]
Conjunctivitis [3]	1.0 [0.8; 1.2]
Dizziness [3]	16.7 [13.1; 20.2]

## EPIDEMIOLOGY OF RESPIRATORY SYNCYTIAL VIRUS

The primary source of RSV infection in older adults are frequently their grandchildren in the community and staff in nursing care homes [16^▪^]. Regardless of the region, the variations in RSV season onset, duration, and offset are relatively consistent year-on-year [5,17]. RSV outbreaks have a consistent duration, averaging 4.6–4.8 months. In temperate areas, RSV epidemics predominantly occur in the winter, but they usually precede influenza outbreaks by about 0.3 months. The influenza season, by contrast, tends to be shorter in temperate zones, averaging around 3.8 months, but extends to 5.2 months in the tropics. RSV epidemics typically start in tropical regions around July, with the onset delayed until January in high-latitude areas. Sub-tropical areas display more varied seasonality with peaks at different time of the year depending on the region. The dominant RSV subtype in circulation does not affect the epidemic's timeline or span [18^▪^].

## CHALLENGES TO IDENTIFYING MORBIDITY BURDEN IN OLDER ADULTS

Estimating the RSV burden in older adults is challenging. Similar symptoms between RSV and other respiratory viral infections, coupled with a lack of awareness and access to timely testing by older adults, lead to underestimations of RSV morbidity burden in older adults. Many studies rely on RT–PCR testing of nasopharyngeal swabs from upper respiratory tract, which may not be representative of the LRTI. Older adults often show diminished viral presence, especially if tested late, and generally have lower viral concentrations than children [44]. Broadening testing to include saliva, sputum, and serology have proven more effective [10^▪▪^]. A recent study in US has shown that RSV prevalence by nasopharyngeal swab alone was 1.8% but increased to 4.5% when saliva (and sputum) was added [10^▪▪^]. These data were utilized in the recent meta-analysis by Li *et al.*[8^▪▪^] to report that the annual estimates RSV hospitalizations in adults ≥65 years industrialized countries could be as high as 787 000 (460–1347). Similarly, the in-hospital annual mortality in this region could be as high as 47 000. Community-based studies provide a varied picture compared to inpatient studies (Tables 2 and 3). While community-based studies provide valuable insights into the range of prevalence/incidence of RSV-ARI in the general older adult population, hospital-based studies offer more precise data on the hospitalization rate, in-hospital deaths, and healthcare impact of RSV.

**Table 2:** RSV burden among older adults in community studies

Settings	Population	Regions	RSV proportion positive	RSV incidence rate	Proportion hospitalized among RSV positive
Community	≥60 years		3.4% to 8.8% [1–7]		
	≥65 years	Industrialized countries		600.7 cases per 100 000 (95% CI: 100.4–3100.5) person-years [8^▪▪^]	
	≥60 years, RSV-positive				0% to 19.5% [2–4]
Outpatients	≥60 years		5.2% to 14.9% [7, 9–16]	500.9 to 2300.2 per 100 000 person-years [17,18^▪^]	
	≥60 years, RSV-positive				11.9% [10]
Emergency department	≥65 years			300.3 (95% CI: 1100.7–9000.8) per 100 000 person-years [19]	

CI, confidence interval; RSV, respiratory syncytial virus.

**Table 3:** RSV burden among older adults in inpatient studies

Settings	Population	Regions	RSV hospitalization rate	RSV in hospital case fatality rate
Inpatients	≥65 years	Industrialized countries	157 (95% CI: 98–252) RSV infections per 100 000 person-years [20]	6.1% (95% CI: 3.3–11.0) [20]
	≥65 years	Industrialized countries	100 (95% CI: 50–210) RSV infections per 100 000 person-years [8^▪▪^]	1.6% (95% CI: 0.7–3.8%) [8^▪▪^]
	≥65 years	Developing countries	30 (95% CI: 10–70) RSV infections per 100 000 person-years [8^▪▪^]	9.1% (95% CI: 2.6–31.8%) [8^▪▪^]
	≥60 years	Industrialized countries		7.1% (95% CI: 5.4–9.3) [21]

CI, confidence interval; RSV, respiratory syncytial virus.

## MORBIDITY BURDEN

### Community burden

Very few community-based studies have reported RSV disease burden in older adults. These are largely from industrialized countries. A meta-analysis by RESCEU investigators identified five studies (with a clear denominator population at risk) from industrialized countries in older adults and reported that the pooled estimate of RSV related acute respiratory infections (ARI) incidence rate was 600.7 [95% confidence interval (CI): 100.4–3100.5] cases per 100 000 person-years [19] (Table 2). Other studies, without a clear denominator population at risk have reported the proportion of older adults with ARI cases testing positive for RSV in community settings; these are highly variable and range from 3.4% to 8.8% depending on the study settings [20–26]. The proportion of ARI cases testing positive for RSV in community and subsequently hospitalized ranged from 0% to 19.5% [21–23]. There is a clear age dependent increase in incidence of RSV-ARI in older adults. The incidence rate of RSV-ARI in older adults per 1000 per year varies by age group: for those aged ≥65 years it ranges from 0.7 to 151.1, for ≥70 years it ranges from 1.6 to 175.0, for ≥75 years it ranges from 6.6 to 175.4, and for those aged ≥80 years, the rates span from 0.9 to 259.7 [27]. In a meta-analysis from industrialized countries examining the incidence of RSV-ARI in adults with comorbidities, the annual incidence rate was found to be 3700.6 (95% CI: 20.1–70.3) per 100 000 persons, while the seasonal incidence rate was 2800.4 (95% CI: 11.4–70.9) per 100 000 persons [12]. In a community-based cohort study involving older adults aged 50 years or older in United States across two RSV seasons (2019–2021), the incidence of RSV-positive ARI before the COVID-19 pandemic was found to be substantial at 4800.6 per 100 000 person-years [28^▪^]. No cases were identified during the pandemic RSV season, though cases re-emerged in the summer of 2021.

Studies in industrialized countries among outpatients aged 60 years and above have reported that RSV positive proportions among those seeking care with ARI varied from 5.2% to 14.9% [26,29–36]. Studies in United States where a reliable population denominator could be estimated have reported an incidence rate of 500.9 to 2300.2 per 100 000 person-years for RSV-ARI in outpatient settings [37,38]. About 11.9% of these RSV-positive older adults in outpatient settings were subsequently hospitalized [30]. In two studies conducted in United States and a multicentre European study, the RSV positive proportions across age groups were relatively consistent [30,31]. Those aged 60–64 years had a proportion of 10% [30]. For the 60–74 years age group, proportions were recorded at 5.18% and 10.78% [30,31]. In the same studies, individuals aged 75 years and above, 8.4% and 11.3% of those with ARI tested positive for RSV [30,31]. In a study conducted in the United States in emergency departments, those aged 65 and over had an RSV incidence rate of 330.9 per 100 000 person-years (95% CI: 110.7–9000.8).

## HOSPITAL BURDEN

There is substantial variability in the estimates for RSV hospitalizations in older adults. The RESCEU investigators reported a pooled hospitalization rate of 157 (95%CI 98–252) per 100 000 persons per year for industrialized countries [8^▪▪^] (Table 3). This translates to about 356 000 (222–572) hospitalizations in industrialized countries in 2019. Another meta-analysis by Savic *et al.*[39] estimated that the hospitalization attack rate for RSV-ARI in older adults in industrialized countries was 0.15% and this translates to about 466 000 (302–720) hospitalizations in industrialized countries in 2019. They estimate about 274 000 (177–423) and 109 000 (71–168) hospitalizations in Europe and USA in 2019. By contrast, Osei-Yeboah *et al.*[40^▪▪^] utilized RSV hospitalization data from RESCEU studies which included multiyear national hospitalization data linked to laboratory reports to develop modelled estimates for Europe and reported 145 000 hospitalizations in older adults in 27 EU countries and the UK. Conversely, RESCEU investigators reported in developing countries, the RSV hospitalization rate for this age group was 30 (95% CI: 10–70) per 100 000 person-years based on data from six studies [19]. This translates to about 109 000 (45–266) hospitalizations in developing countries. In Europe, the average annual number of hospitalizations for the age groups 65–74 years, 75–84 years, and ≥85 years were 32 679 (95% CI: 27 594; 37 764), 74 519 (95% CI: 69 923; 79 115), and 37 904 (95% CI: 32 444; 43 363), respectively [40^▪▪^]. The hospitalization rate of RSV-ARI in older adults per 100 000 per year increases with age: for those aged ≥65 years, the rate ranges from 10 to 320; for ≥70 years, it varies from 10 to 460; for ≥75 years, it spans from 0.0 to 710; and for those aged ≥80 years, it's between 0.0 and 1410 [27]. A meta-analysis from industrialized countries reported that the odds ratio for RSV-ARI hospitalization in patients with comorbidities (asthma, CHF, COPD, diabetes, and immunocompromised) compared to those without was 4.1 (95% CI: 1.6–10.4). [12]. Moyes *et al.*[41] from South Africa reported that the hospitalization rates for RSV-ARI in adults with HIV (in 2012) were 400.8, 200.0, and 200.0 per 100 000 persons/year for age groups ≥65 years, 45–64 years, and 18–44 years, respectively. Falsey *et al.*[42] from the United States reported a hospitalization rate of 1300.2 per 100 000 persons/year for adults aged ≥65 with chronic heart failure or chronic obstructive pulmonary diseases due to RSV-ARI.

## MORTALITY BURDEN

In industrialized countries, the in-hospital case fatality rate (hCFR) attributed to RSV infections for older adults shows substantial variation, with estimates from three meta-analyses ranging from 1.6% (95% CI: 0.7–3.8) [19] to 6.1% (95% CI: 3.3–11.0) [8^▪▪^] and 7.1% (95% CI: 5.4–9.3) [39]. The latter estimate (7.1%) from of Savic *et al.*[19] study translates to about 33 000 (16–67) deaths. In developing countries, the hCFR for this age group was 9.1% (95% CI: 2.6–31.8). This translates to about 10 000 (2–46) deaths, largely driven by the low number of RSV hospitalizations in this region. Among older adults, the hCFR of RSV-ARI varies with age: 4.6% for those aged 60–74 years and 7.3% for those aged ≥75 years [43]. In a systematic review examining the burden of RSV in older adults with comorbidities in both industrialized and developing countries, the hCFR for RSV-ARI in adults with any comorbidity stood at 11.0% (95% CI, 6.8; 17.9) [12].

## COMPARISON WITH INFLUENZA BURDEN

Several reports have shown that while RSV disease burden is well recognized in young children, it is under appreciated in older adults. Therefore, seasonal influenza that causes substantial morbidity and mortality in older adults, offers a useful anchor point for comparing disease burden and impact on healthcare systems. Studies have shown that in general RSV disease burden may be slightly lower or even comparable to influenza. For example, Falsey *et al.*[20] analyzed data over four consecutive winters from their cohort in Rochester, New York and reported that although RSV infection generated fewer clinic visits than influenza (17% and 29% in healthy and high risk older adults respectively, compared to 42% and 60% respectively for influenza A), use of healthcare services by high-risk adults was similar in both groups (9% and 16% for emergency room visits and hospitalizations respectively for RSV compared to 16% and 20% respectively for influenza A). In the hospitalized cohort, RSV and influenza A infections resulted in similar lengths of stay, rates of use of intensive care (15% and 12% respectively), and mortality (8% and 7%, respectively). A timeseries modelling study from the United Kingdom using data from the Public Health England (PHE) weekly pathogen surveillance for influenza and RSV, the Clinical Practice Research Datalink (CPRD), the Hospital Episode Statistics (HES), and the Office of National Statistics (ONS) databases for the period 1997 to 2009 showed that the RSV: Influenza ratio for GP episodes and hospitalizations for respiratory disease in adults ≥65 years was 1.6 : 1 and 0.8–0.9 : 1 [45]. The antibiotic prescriptions ratio for RSV and influenza was 2 : 1. A recent timeseries including data over a 20 year period from US reported that mean excess respiratory and circulatory deaths associated with RSV in adults ≥65 years was 12604 (95% CI 11808–139999) compared to 14496 (13465–15528) for influenza [46]. In the UK, ratio of deaths due to respiratory and cardiorespiratory disease in adults ≥65 years from RSV and influenza was broadly comparable (0.9 : 1) [45].

## COSTS RELATED TO RESPIRATORY SYNCYTIAL VIRUS HOSPITALIZATIONS OR RESPIRATORY SYNCYTIAL VIRUS ILLNESS IN OLDER ADULTS

A community study across Belgium, the UK, and the Netherlands for two RSV seasons highlighted varied direct mean costs for GP visits per RSV episode: €11.7 (median and IQR: 3.4; 0; 12.2) from the patient's perspective, €14.6 (median and IQR: 0; 0; 23.2) healthcare provider's, and €26.3 (median and IQR: 5.5; 0; 47.3) healthcare payer's. Comparable influenza costs were slightly higher, though interquartile ranges showed substantial overlaps [47^▪^]. Several studies from various regions have evaluated the cost burden and hospitalization duration associated with RSV in older adults. A United States study of 601 patients hospitalized with RSV revealed that 57% were older adults with an average hospitalization cost of $8241 (95% CI: 6957; 9758) and duration of hospitalization for 8 days (95% CI: 7; 9) [48]. This implies an estimated U.S. annual cost burden of $743.9 million (95% CI: 542.2; 945.7) for RSV associated hospitalizations in older adults. Another United States comparison between RSV (579 patients) and influenza (1511 patients) showed that RSV hospitalizations incurred a slightly longer stay of 0.68 days (0.02 to 1.37) and cost $16 034 (14 684; 17 440), compared to $15 163 (14 192; 16 225) for influenza with a nonsignificant difference of $871 (−811–2547) [49]. Additionally, immunocompromised U.S. RSV patients had longer hospital stays (geometric mean 7.3 vs. 5.4 days) and higher costs (geometric mean $66 476.1 vs. $29 316.2) compared to their nonimmunocompromised counterparts and similar patterns were noted for influenza [50]. In China, RSV patients (median LOS: 14.0 days, IQR: 10.0; 23.0 days) had a longer stay (*P* value < 0.001) but lower cost (*P* value < 0.001) ($2919.1, IQR: $1172.1–15627.4) than influenza patients (median LOS: 10.0 days, IQR: 8.0; 14.0 days; cost: $3367.5, IQR: $1896.1 to $10767.0) [51]. A New Zealand study estimated the yearly RSV hospitalization cost at $525 137.85, translating to an average of $3053.13 per hospitalization [52]. They reported that the median length of hospital stay for RSV positive patients was 4 days (IQR: 2; 6 days). A Korean study reported an average RSV hospital stay of 20.4 days (±33.6) with a median cost per each admission of $2933.17 (IQR: $1748.26; $6339.93) [53]. In Canada in multiple care settings, RSV-attributable ARI healthcare costs varied based on age, with the 65–79 age group incurring $1491 (± $5675) and those aged 80 and above $2878 (±$7230) per episode [54].

## LOOKING FORWARD

Considerable progress was made in developing RSV vaccines for older adults [14^▪▪^,^15^^▪▪^]. The United States Food and Drug Administration (USFDA) and European Medicines Agency (EMA) recently approved two RSV vaccines for this age group: RSVpreF (unadjuvanted bivalent RSV vaccine with F protein in prefusion conformation) and RSVPreF3 (adjuvanted monovalent RSV vaccine with F protein in prefusion conformation), both of which demonstrated very high efficacy rates along with durable protection for at least 2 years. The RSVPreF3 vaccine has been also licensed in Japan and Canada. RSVpreF, tested on 34 284 participants enrolled between August 31, 2021, and July 14, 2022, showed 66.7% (96.6% CI: 28.8; 85.8) efficacy against RSV-associated LRTI with at least two symptoms and 85.7% (96.6% CI: 32.0; 98.7) with at least three symptoms at the end of the first season [15^▪▪^]. RSVPreF3, evaluated on participants enrolled in 2021–2023, demonstrated an efficacy of 74.5% (95% CI: 60.0; 84.5) against RSV-associated LRTI over combined seasons 1 and 2. In season 1, the efficacy against RSV-associated LRTI was 82.6% (95% CI: 57.9; 94.1) and 87.5% (95% CI: 58.9; 97.6) against RSV-associated medically attended LRTI. In the season 2, the efficacy against RSV-associated LRTD was 56.1% (95% CI: 28.2; 74.4) [14^▪▪^,^55^^▪▪^]. Both vaccines demonstrated similar efficacies in adults ≥ 75 years as well as those with comorbidities. Concerns emerged regarding certain side effects, such as Guillain–Barré syndrome and atrial fibrillation, observed postvaccination in trials of both vaccines [55^▪▪^]. Based on the safety and efficacy data as well as current disease burden data, the US Centres for Disease Control and Prevention (CDC) Advisory Committee on Immunization Practices (ACIP) has recommended single dose of RSV vaccine in adults ≥60 years based on shared clinical decision-making considering individual risk factors, health status, and preferences [55^▪▪^]. The ACIP estimates that in the US alone, every one million vaccine doses given over two seasons would prevent 41 000–44 000 outpatient visits, 3190 to 3460 hospitalisations and 155 to 167 deaths in adults ≥ 60 years of age [55^▪▪^]. In the UK, the Joint Committee on Vaccination and Immunisation (JCVI) has recommended a single dose of RSV vaccine in all adults ≥75 years. These developments, along with the promising efficacy of mRNA-1345 against RSV-related LRTI, represent an exciting opening chapter in RSV prevention for the elderly [56–58].

## CONCLUSION

The prevalence and severity of RSV among older adults, particularly those aged 60 and over, are becoming increasingly recognized, with several studies highlighting its widespread nature both in industrialized and developing countries. The disease burden is likely to be comparable to seasonal influenza. The frail elderly and those with multimorbidities are at substantial risk of severe disease (including prolonged hospitalization) and death. As a result, RSV vaccines displaying efficacies of up to 94.1% with duration of protection extending at least two seasons offer hope. However, concerns about possible side effects require continued monitoring and rigorous research to ensure the safe and effective management of RSV among the older adults.

## Acknowledgements

*None.*

*Author contributions: Both authors contributed equally to the article. Both authors approve this manuscript.*

### Financial support and sponsorship

*None.*

### Conflicts of interest

*H.N. reports grants outside the submitted work from the Innovative Medicines Initiative, WHO, the National Institute for Health Research, Pfizer and Icosavax; and personal fees from the Bill & Melinda Gates Foundation, Pfizer, GSK, Merck, Abbvie, Janssen, Icosavax, Sanofi, Novavax, outside the submitted work.*

## REFERENCES AND RECOMMENDED READING

Papers of particular interest, published within the annual period of review, have been highlighted as:

▪ of special interest
▪▪ of outstanding interest
